# Probiogenomic analysis of *Limosilactobacillus fermentum* SD7, a probiotic candidate with remarkable aggregation abilities

**DOI:** 10.1016/j.heliyon.2025.e42451

**Published:** 2025-02-03

**Authors:** Thunchanok Yaikhan, Monwadee Wonglapsuwan, Nuntiya Pahumunto, Natakorn Nokchan, Rawee Teanpaisan, Komwit Surachat

**Affiliations:** a Department of Biomedical Sciences and Biomedical Engineering, Faculty of Medicine, Prince of Songkla University, Hat Yai, Songkhla, 90110, Thailand; b Division of Biological Science, Faculty of Science, Prince of Songkla University, Hat Yai, Songkhla, Thailand; c Research Center of Excellence for Oral Health, Faculty of Dentistry, Prince of Songkla University, Hat Yai, Songkhla, Thailand; d Department of Oral Diagnostic Sciences, Faculty of Dentistry, Prince of Songkla University, Hat Yai, Songkhla, Thailand; e Medical Science Research and Innovation Institute, Prince of Songkla University, Hat Yai, Songkhla, 90110, Thailand; f Translational Medicine Research Center, Faculty of Medicine, Prince of Songkla University, Hat Yai, Songkhla, 90110, Thailand

**Keywords:** *Limosilactobacillus fermentum*, Probiotic, Immunomodulatory, Whole genome analysis, Aggregation ability

## Abstract

*Limosilactobacillus fermentum* has gained recognition as a probiotic due to its immunomodulatory properties. In this study, we characterized *L. fermentum* SD7, which was isolated from the human oral cavity. The genome of *L. fermentum* SD7 was approximately 2.27 Mb in size, with a 51.1 % GC content. Using comprehensive genome analysis, we compared the genome of *L. fermentum* SD7 with 153 available genome sequences of *L. fermentum* strains and categorized the 154 strains into six distinct clades based on core gene single nucleotide polymorphisms. Among the 12,598 orthologous proteins, we identified 910 core genes and 10,169 accessory genes. Our analysis revealed a close similarity between *L. fermentum* SD7, FS-10, and L13. In addition, *L. fermentum* SD7 genome contains four strain-specific putative CRISPR-associated genes and lacks antimicrobial resistance and virulence genes. Importantly, we identified 27 genes in *L. fermentum* SD7 genome that are linked to aggregation ability, which is supported by our probiogenomic analysis. This aggregation ability is considered crucial for the probiotic efficacy of *L. fermentum* SD7. These findings provide a comprehensive understanding of the genetic composition of *L. fermentum* and its potential probiotic properties, identifying *L. fermentum* SD7 as a promising probiotic candidate for use in the food and healthcare industries.

## Highlights

• *L. fermentum* SD7 displays significant aggregation ability, crucial for its probiotic efficacy from probiogenomic analysis.
• 27 genes in *L. fermentum* SD7 potentially regulate aggregation ability crucial for probiotic efficacy.
• No antibiotic resistance genes found in *L. fermentum* SD7.
• SD7 strain-specific putative CRISPR-associated genes identified.
• Promising probiotic for food and healthcare industries due to genetic insights.

## 1 Introduction

Probiotics are defined as living microorganisms that, when consumed in sufficient quantities, provide health benefits to the host. They are commonly utilized in both medical treatments and various food items. Among these probiotics, certain strains of *Limosilactobacillus fermentum*, previously classified as *Lactobacillus fermentum* [1,2], are extensively employed. *L. fermentum* represents a Gram-positive bacterium typically characterized by its morphology as non-spore-forming rods or coccobacilli [3], and exhibit fermentative, aerotolerant, and acidophilic properties. Among lactic acid bacteria (LAB), *Limosilactobacillus* species are notable for their complex nutritional requirements, which include a variety of carbohydrates, amino acids, peptides, fatty acid esters, salts, nucleic acid derivatives, and vitamins [4].

*L. fermentum* is widely distributed in various environments, such as the mucosal membranes of humans and animals, plant surfaces, manure, sewage, and fermented or spoiled foods [5]. Its ability to metabolize sugars and produce lactic acid has made it a common starter culture in the food industry, particularly for yogurt, cheese, and fermented vegetables [6]. LAB are recognized for their health benefits and functional properties, which have been scientifically validated, leading to their classification as “Generally Recognized As Safe” (GRAS) by regulatory authorities [7]. Probiotic bacteria are known to offer a range of health benefits, including the regulation of gut microbiota and the prevention of gastrointestinal disorders such as irritable bowel syndrome, inflammatory bowel disease, and antibiotic-associated diarrhea [[8], [9], [10], [11]].

Probiotic efficacy relies on the presence of critical functional characteristics. These properties include the ability to survive harsh gastrointestinal conditions such as low pH and bile salts, adherence to intestinal epithelial cells, production of beneficial enzymes, and immunomodulatory effects [12]. *L. fermentum* has been shown to exhibit antimicrobial activity by producing bacteriocins and other antimicrobial metabolites that inhibit the growth of pathogens [13]. Additionally, probiotic strains, including *L. fermentum*, are evaluated for their safety, which entails the absence of antimicrobial resistance genes (ARGs), virulence factors, and harmful enzymes such as β-glucuronidase, which is associated with carcinogen conversion [13,14]. The production of enzymes such as β-galactosidase, which aids in lactose hydrolysis, is another valuable probiotic property, especially in the food industry where it is used to improve the texture and digestibility of dairy products [15].

10.13039/100014337Furthermore, probiotic bacteria can enhance host immunity by interacting with the gut microbiome to produce metabolites such as short-chain fatty acids and complex sugars, which support proper organ function, nutrient absorption, and immune modulation [16,17]. Specific surface proteins in LAB, such as sortase and LPXTG motifs, are involved in adhesion to the host intestinal mucosa, facilitating colonization and persistence in the gastrointestinal tract. This adherence capability is a critical probiotic property as it allows probiotics to exert their beneficial effects over extended periods [[18], [19], [20]].

Another important probiotic characteristic of *L. fermentum* is its aggregation ability, which enhances its efficacy. Aggregation includes the ability of bacterial cells to cluster together (auto-aggregation) or bind to pathogens (co-aggregation), thus inhibiting pathogen colonization and supporting probiotic adherence in the gut [21]. Previous studies have explored surface pathogen aggregation proteins and single-chain antibodies in *Limosilactobacillus* strains to enhance their attachment to intestinal epithelial cells (IECs), thereby increasing their colonization potential both *in vitro* and *in vivo* [[22], [23], [24], [25]]. However, the exact mechanisms of aggregation, the optimal conditions for epithelial cell interaction, and the processes required for effective probiotic aggregation remain largely unknown.

Metagenomic studies have revealed that *Limosilactobacillus* species are dominant members of the microbial consortia in the human gut, vagina, and oral cavity, contributing to various health benefits [[26], [27], [28]]. This study explores the probiotic potential of *L. fermentum* SD7, a strain isolated from the human oral cavity. Previous research has demonstrated its strong internalization ability and effectiveness in inhibiting pathogenic bacteria through exclusion, competition, and displacement [29]. Furthermore, *L. fermentum* SD7 exhibited significant adhesion to intestinal epithelial cells and antimicrobial activity, making it a promising candidate for probiotic applications. Despite these beneficial properties, genomic data for this strain is limited. To gain a deeper understanding of its probiotic mechanisms, we conducted whole-genome and comparative analyses, focusing on genes associated with its aggregation ability and other probiotic functions.

## 2 Materials and methods

### 2.1 Bacterial strain isolation, genomic DNA extraction, and whole genome sequencing

*L. fermentum* SD7 was isolated from the saliva of children in the previous study [30]. To culture this strain, a single colony was streaked onto a De Man, Rogosa, and Sharpe (MRS) agar (Difco BD, NJ, USA). The colonies were then streaked onto MRS agar plates and incubated anaerobically at 37 °C for 24 h. The bacterial genomic DNA was then extracted using the TIANamp Bacterial DNA Kit (Tiangen Biotech Co. Ltd., Beijing, China) according to the manufacturer's protocol. The concentration and quality of the extracted DNA were measured using a NanoDrop 2000c spectrophotometer (Thermo Fisher Scientific, USA), while its integrity and purity were checked via agarose gel electrophoresis. The extracted DNA was subsequently utilized for library preparation and sequenced on the DNBSEQ-G50 platform (BGI, China), generating 150-bp paired-end reads totaling 1 Gbp in FASTQ format.

### 2.2 Sequence analysis

The raw reads were processed using the BacSeq v1.0.0 pipeline [31] for assembly and annotation. This pipeline streamlines the analysis by integrating multiple tools, including SPAdes v3.15.5 [32] for assembly, Prokka v1.12 [33] for functional annotation, tRNAscan-SE [34] for tRNA gene prediction, and RNAmmer [35] for rRNA gene identification. The draft genome assembly was subsequently refined by scaffolding with RagTag v2.1.0 [36], utilizing a closely related complete genome (*L. fermentum* IFO 3956, GCA_000010145.1). ABRIcate was used to investigate antimicrobial resistance and virulence genes against the CARD and VFDB databases, respectively [37,38]. Plasmid screening was performed using an established *in silico* method [39,40], and clustered regularly interspaced short palindromic repeats (CRISPRs) were screened for using CRISPRFinder [41]. Proksee was used to visualize the circular genomes, and Phigaro was used to identify and annotate prophage regions [[42], [43], [44]].

To identify genes associated with internalization, adhesion, and aggregation, we conducted a comprehensive analysis using genes obtained from relevant studies [4,45,46]. To identify homologous genes and their functional roles related to probiotic features in the target genome, we employed the Basic Local Alignment Search Tool (BLAST) for nucleotide sequence alignment. To ensure reliable results, we applied an *E*-value threshold of 1 × 10⁻^20^ and required a minimum identity of 70 %. Furthermore, we used BLASTp to identify genes involved in the production of ribosomally synthesized and post-translationally modified peptides (RiPPs) and bacteriocins by comparing our sequences with the Bagel database. Relevant gene clusters were further analyzed and visualized using the Bagel4 webserver [47]. Additionally, antiSMASH 6.0 was used to detect and examine gene clusters associated with secondary metabolite biosynthesis [48].

### 2.3 Comparative genome analysis

We obtained a total of 162 *L. fermentum* genome sequences from the NCBI Reference Sequence Database (RefSeq). Subsequently, all genome sequences underwent evaluation for completeness by BUSCO v5.6.1 [49] using lactobacillales_odb10.2019-04-24 database. Genome sequences with completeness levels lower than 97 % were excluded from further analysis. All remaining 153 genomes, along with the SD7 genome, were re-annotated using the same pipeline, Prokka 1.14.6 [33], to avoid bias from gene calling using different tools. Core, accessory, and unique protein families were determined using Roary with a 95 % BLASTp threshold and default settings [50]. We identified 910 core genes for further analysis. SNPs of core genes were then called using snp-sites 2.5.1 [51]. A phylogenetic tree was then constructed using the neighbor-joining method in Geneious software [52], with 1000 bootstrap repetitions for validation. Tree visualization was performed using iTOL v6.8.1 [53].

Furthermore, we compared *L. fermentum* SD7 with the most closely related probiotic strains *L. fermentum* FS10 and *L. fermentum* L13 to visualize similarities among these genomes using Proksee [43], and OrthoANI for average nucleotide identity analysis [54]. Additionally, Clusters of Orthologous Genes (COGs) were identified using PanExplorer webserver [55]. Statistical analysis was evaluated using IBM SPSS Statistics version 26, USA. To compare the proportion of COG number of each strain, Fisher's exact test was used.

## 3 Results

### 3.1 Genome sequence and plasmid identification of *L. fermentum* SD7

**Fig. 1:**
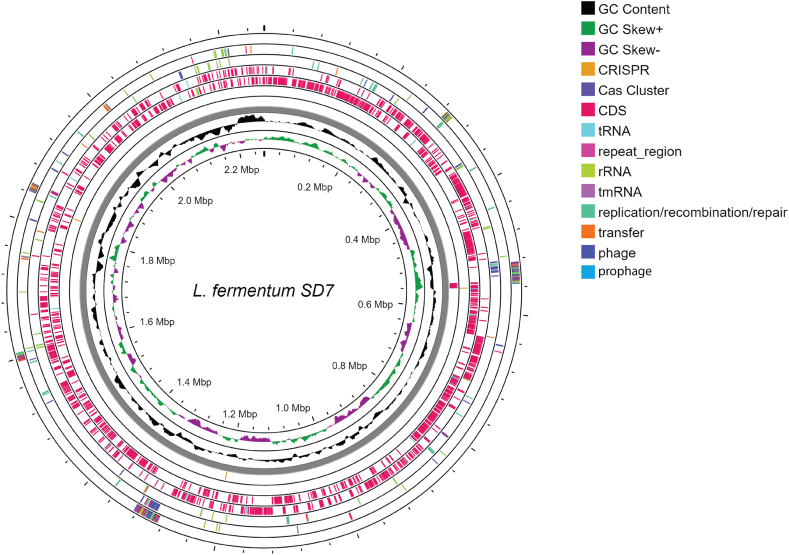
Genome map of *L. fermentum* SD7 generated using Proksee. The map provides a comprehensive overview of the genome, with pink arrows representing coding sequences (CDSs) and gray arrows representing contigs. The black plot indicates the GC content. The green and magenta plots represent the CG-skew in positive and negative directions, respectively.

The genome of *L. fermentum* SD7 was sequenced using the MGISEQ-2000 platform (BGI, Beijing, China). The genome size was approximately 2.27 Mb, with a GC content of 51.1 %. Sequencing yielded 27 contigs, 13 of which were identified as plasmid contigs (Supplementary Table 1). Genome annotation using Prokka successfully identified 2280 coding sequences and 55 RNA molecules, including 2 rRNAs, 52 tRNAs, and 1 tmRNA (transfer-messenger RNA). The circular graphical representation of the *L. fermentum* SD7 genome is shown in Fig. 1. We utilized Proksee to classify 841 of the 2280 coding sequences into clusters of COGs. Notably, among the identified coding genes, 948 (41.58 %) were annotated as hypothetical proteins. Further genomic analysis revealed two clusters of CRISPR-Cas (CRISPR-associated) genes and three prophage gene loci encoded in the SD7. The assessment of ARGs and virulence genes using ABRIcate revealed that *L. fermentum* SD7 contains no detectable ARGs or virulence genes, supporting its designation as a GRAS-status probiotic. Moreover, we identified genes in *Lactobacillus fermentum* SD7 that potentially associated with various stress tolerance mechanisms, including acid tolerance, bile salt tolerance, and cold shock responses, indicating their potential probiotic properties. A comprehensive overview of these genes and their functions is present in Supplementary Table 2.

For plasmid contigs, there were six plasmids that contain genes pivotal for this strain. Contig P1 includes genes for chromosome partitioning, transposition, and resistance to daunorubicin/doxorubicin. Contig P2 contains genes for transposition, transcriptional regulation, oxidation-reduction reactions, recombination, metal transport, and DNA topology control. Contig P3 harbors genes for a putative protein, vitamin B12 import, and redox reactions. Contig P4 encompasses genes for amino acid biosynthesis, peptide transport, and DNA metabolism. Contig P5 includes genes for sporulation inhibition, protein processing, and transposition. Contig P6 encodes enzymes for amino acid biosynthesis, transcriptional regulation, and various transporter and kinase activities (Supplementary Table 3). These findings may highlight the genetic diversity and functional versatility of the plasmids in *L. fermentum* SD7, potentially contributing to its adaptability and ecological success.

### 3.2 Comparative genomic analysis

**Fig. 2:**
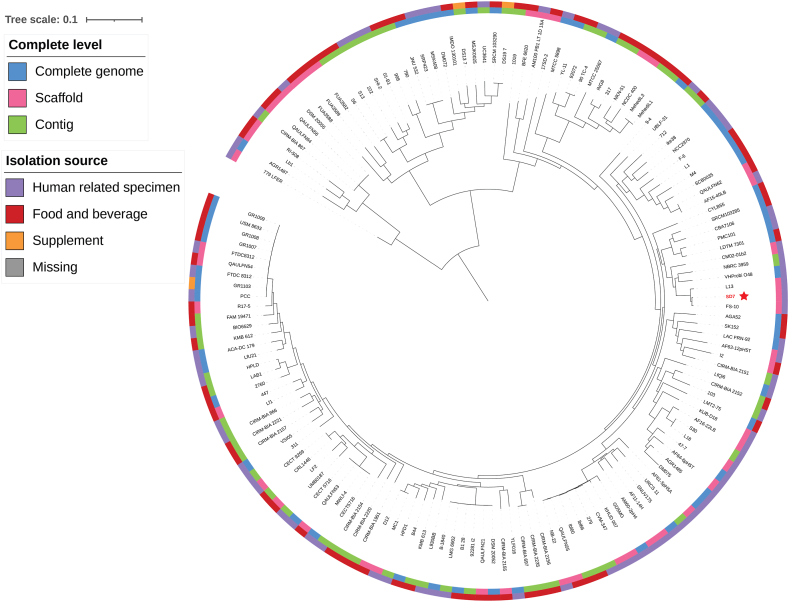
The phylogenetic tree depicts the positioning of *L. fermentum SD7* (highlighted with a red star) in comparison to 153 other strains of *L. fermentum*. The tree was generated based on core-genome SNPs using Geneious software, employing the neighbor-joining method for reliability. Visualization and annotation were accomplished using iTOL v6.8.1.

We conducted comparative genomic analyses to examine the distinct characteristics of *L. fermentum* SD7 and other *L. fermentum* strains. Using core gene single nucleotide polymorphism (SNP) analysis across 154 strains (Supplementary Table 4), we identified six distinct clades within *L. fermentum*, placing SD7 in clade 4 alongside strains FS-10 and L13, both isolated from human feces. Unlike FS-10 (1,785,017 bp with 87 contigs) and L13 (1,947,210 bp with 147 contigs), SD7 exhibited a larger genome size with a higher plasmid content, indicating potential for enhanced environmental adaptability (Fig. 2).

**Fig. 3:**
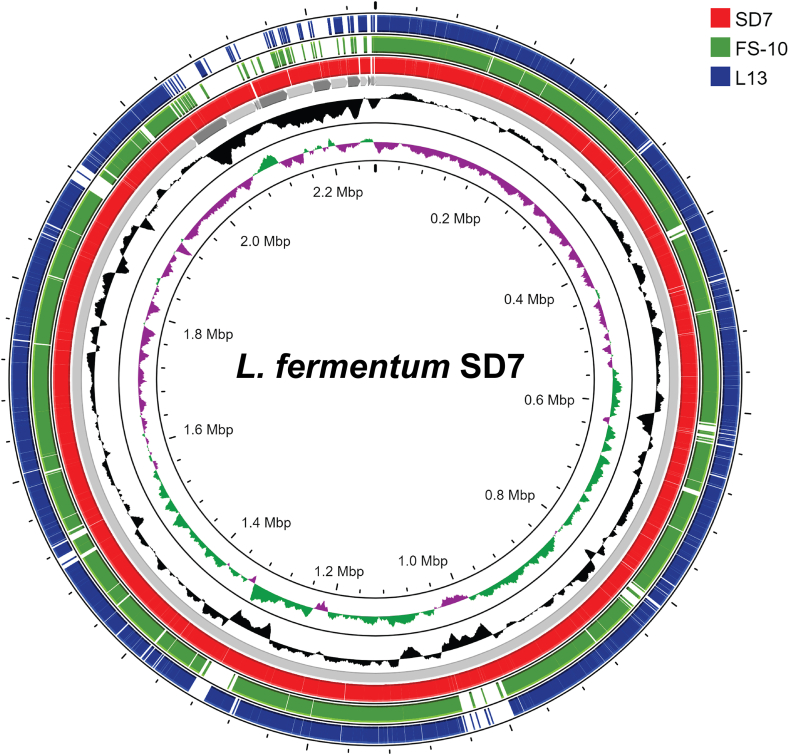
Comparative whole-genome analysis of *Limosilactobacillus fermentum* SD7 with strains FS-10 and L13 generated by Proksee. From outermost to innermost, the blue circle indicates the genome of *L. fermentum* strain FS10, the green circle indicates the *L. fermentum* L13 genome, and the red circle represents strain SD7. White gaps indicate the absence of coding regions among these three strains. The innermost black plot indicates GC content, while the green and magenta plots show the CG-skew in positive and negative directions, respectively.

A comparative circular genome analysis (BLAST similarity) demonstrated considerable gene conservation among SD7, FS-10, and L13 (Fig. 3), while orthologous average nucleotide identity (orthoANI) analysis confirmed high sequence similarity (Supplementary Fig. 3). Functional category analysis of strain-specific genes, conducted via the PanExplorer web tool, showed that SD7 harbors unique gene clusters related to stress adaptation, plasmid maintenance, and metabolic versatility. Interestingly, SD7 displayed a greater percentage of strain-specific genes unclassified into COGs (32.4 %), which may indicate novel genes with potential roles in adaptation and functionality.

**Fig. 4:**
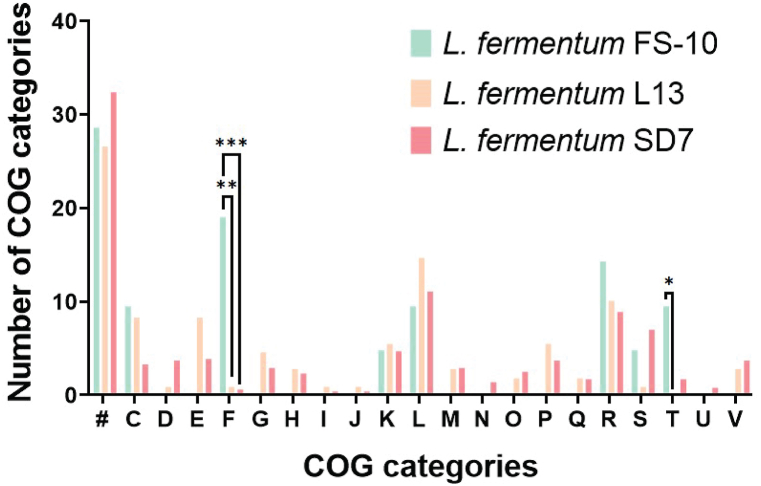
Presents the distribution of COG (Clusters of Orthologous Groups) functional categories among *L. fermentum* strains SD7, FS10, and L13. Each category is denoted by a specific letter: # indicates “Not in COGs”; C represents “Energy production and conservation”; D stands for “Cell cycle control, cell division, and chromosome partitioning”; E corresponds to “Amino acid transport and metabolism”; F refers to “Nucleotide transport and metabolism”; G is for “Carbohydrate metabolism and transport”; H represents “Coenzyme metabolism”; I stands for “Lipid metabolism”; J indicates “Translation”; K is for “Transcription”; L corresponds to “Replication, recombination, and repair”; M denotes “Cell wall/membrane/envelope biogenesis”; N refers to “Cell motility”; O represents “Post-translational modification, protein turnover, and chaperone functions”; P stands for “Inorganic ion transport and metabolism”; Q corresponds to “Secondary structure”; R is for “General functional prediction only”; S indicates “Function unknown”; T stands for “Signal transduction”; U represents “Intracellular trafficking and secretion”; and V is for “Defense mechanisms.” Statistical comparisons across the three strains were performed using Fisher's exact test, with significance levels indicated as ∗∗∗P < 0.001, ∗∗P < 0.01, and ∗P < 0.05.

Among classified genes, SD7's specific genes were predominantly associated with categories such as replication, recombination, and repair (11.1 %) and general functional prediction (8.9 %). In contrast, FS-10 and L13 were enriched in categories like nucleotide transport and metabolism (19.0 % in FS-10) and signal transduction (p = 0.025 between FS-10 and L13). These findings suggest that SD7's unique genomic profile, particularly its enhanced genetic capacity for DNA maintenance and repair, could support its resilience and persistence in diverse environments, which may contribute to its potential as a robust probiotic strain (Fig. 4).

### 3.3 Pan-genome analysis of *L. fermentum*

**Fig. 5:**
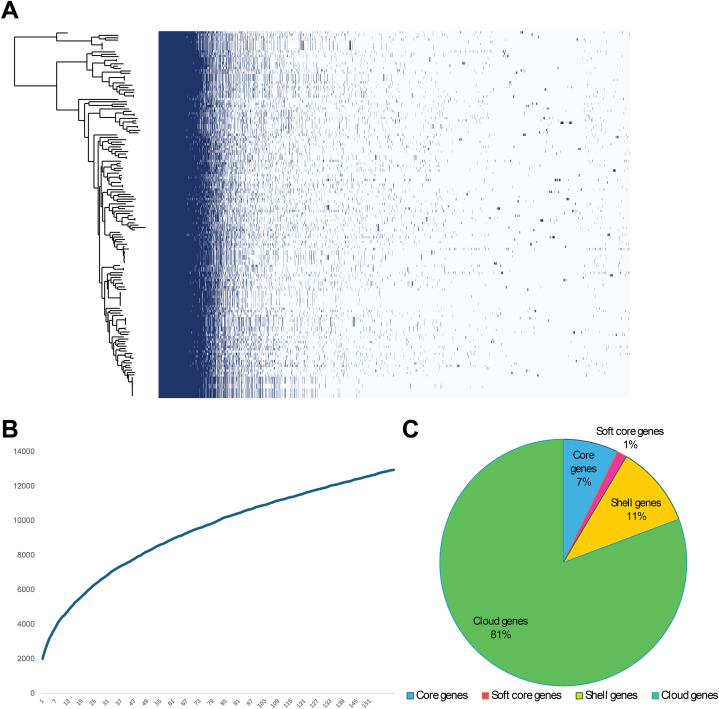
*L. fermentum* pan-genome analysis using Roary software. (A) Core and accessory genes were set as 99–100 % and 15%–95 %, respectively. The evolutionary tree is shown on the left. The presence/absence of genes is graphically represented on the right. The heat map shows gene presence (dark blue) or absence (light blue) of 12,939 orthologous proteins set; the histogram indicates the distribution of how many genomes were found within each gene. (B) The pan genome of the 154 *L. fermentum* genomes. (C) The percentage of genes in the pan-genome.

Pan-genome analysis evaluated the diversity among 154 *L. fermentum* genomes by comparing SD7 with all genomes of *L. fermentum* from the RefSeq database. A pan-genome of 12,598 gene clusters, including core (7 %, 910 genes), accessory (81 %, 10,169 genes), shell (11 %, 1354 genes), and soft-core (1 %, 165 genes) gene sets were identified. The core gene set consists of genes that are present in all strains with a sequence identity of over 95 %. Genes that are not found in every strain are classified as accessory genes, while genes unique to a single strain are designated as strain-specific genes. Visualization of the pan-genome analysis (Fig. 5A and C) indicated a high proportion of accessory genes (light blue area) to core genes (dark blue area), which indicates a high level of genetic diversity in this species. The pan-genomes of the 154 *L. fermentum* genomes are represented by black lines in the tree. The number of genes increased continuously with the addition of additional genomes, indicating an open pan-genome for *L. fermentum* (Fig. 5B).

### 3.4 Evaluation of genes associated with adhesion and aggregation in *L. fermentum* SD7

**Fig. 6:**
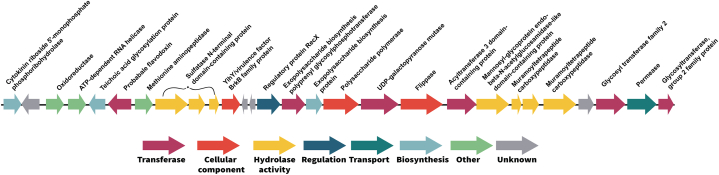
Visualization of the EPS cluster within the *L. fermentum* SD7 genome spans nucleotide numbers 1,068,154 to 1,094,893. Genes within the cluster are color-coded based on their respective functions, as specified in the legend provided below. The gene names and functions, annotated by Uniprot.

The ability of probiotic strains to adhere to host epithelium is attributed to their cell surface proteins. The SD7 strain contains 26 genes putatively encoding aggregation-related proteins (Table 1). Among these, fourteen genes are associated with inner membrane proteins that support SD7 aggregation and adhesion to epithelial cells. Additionally, two genes are linked to lipoprotein production, which plays a vital role in Gram-positive bacteria by promoting cell adhesion and aggregation to host tissue. Three genes regulate adhesive glucan synthesis via the action of glucosyltransferases. Furthermore, four genes regulate adhesin production and are responsible for bacterial attachment to peptidoglycans of the bacterial cell wall. Two fimbria-associated genes, mediating adhesion and aggregation to other bacterial cell surfaces, were also identified. Moreover, a cluster of genes associated with exopolysaccharide (EPS) was identified, aiding in cell–cell or host–cell adhesion (Fig. 6). The biosynthesis of EPSs in LAB involves four key types of functional proteins: regulation, biosynthesis of repeating units, polymerization and chain-length determination, and export. These proteins work together to regulate EPS production in response to environmental signals, synthesize the building blocks of EPS, polymerize them into long chains, and export the synthesized EPS molecules out of the cell [56]. As illustrated in Fig. 6, we identified a 26,739 bp EPS cluster in the SD7 chromosome that plays a pivotal role in EPS biosynthesis. The cluster comprises various types of protein categories, including transferase, cellular component, hydrolase activity, regulation, protein transport, biosynthesis, and other functions (Supplementary Table 5).

**Table 1:** List of genes associated with adhesion and aggregation in *L. fermentum* SD7.

Function	locus_tag	Length (bp)	Gene	Product
Inner membrane	BIKNMBLJ_00183	516	*yagU*	Inner membrane protein YagU
	BIKNMBLJ_00194	681	*ydfK_1*	Putative membrane protein YdfK
	BIKNMBLJ_00208	1359	*ygcS*	Inner membrane metabolite transport protein YgcS
	BIKNMBLJ_00658	711	*ybhL*	Inner membrane protein YbhL
	BIKNMBLJ_01201	1506	*yjeM_1*	Inner membrane transporter YjeM
	BIKNMBLJ_01232	1326	*yhjE_1*	Inner membrane metabolite transport protein YhjE
	BIKNMBLJ_01467	1197	*ydiM*	Inner membrane transport protein YdiM
	BIKNMBLJ_01656	1344	*yhjE_2*	Inner membrane metabolite transport protein YhjE
	BIKNMBLJ_01681	918	*yicL_1*	Putative inner membrane transporter YicL
	BIKNMBLJ_01700	912	*yicL_2*	Putative inner membrane transporter YicL
	BIKNMBLJ_01885	1497	*yjeM_2*	Inner membrane transporter YjeM
	BIKNMBLJ_02034	906	*ydjE_1*	Inner membrane metabolite transport protein YdjE
	BIKNMBLJ_02043	1341	*ydjE_2*	Inner membrane metabolite transport protein YdjE
	BIKNMBLJ_02070	1110	*ydhP*	Inner membrane transport protein YdhP
Lipoprotein	BIKNMBLJ_00735	849	*tpn32*	Membrane lipoprotein TpN32
	BIKNMBLJ_02077	438	*lspA*	Lipoprotein signal peptidase
Glucan synthase	BIKNMBLJ_01313	1497	*gtfA_1*	UDP-N-acetylglucosamine--peptide N-acetylglucosaminyltransferase GtfA subunit
	BIKNMBLJ_01314	1539	*gtfA_2*	UDP-N-acetylglucosamine--peptide N-acetylglucosaminyltransferase GtfA subunit
	BIKNMBLJ_02187	2988	*gtfC*	Glucosyltransferase-SI
Adhesin	BIKNMBLJ_00005	777	*tpiA_1*	Triosephosphate isomerase
	BIKNMBLJ_00303	834	*misCA*	Membrane protein insertase MisCA
	BIKNMBLJ_00705	702	*strA*	Sortase A
	BIKNMBLJ_02137	1323	*eno2*	Enolase 2
Fimbria production	BIKNMBLJ_00720	906	*fimA*	Manganese ABC transporter substrate-binding lipoprotein
	BIKNMBLJ_01294	1455	*mltF*	Membrane-bound lytic murein transglycosylase F

### 3.5 Prediction of gene clusters involved in secondary metabolite synthesis

**Fig. 7:**
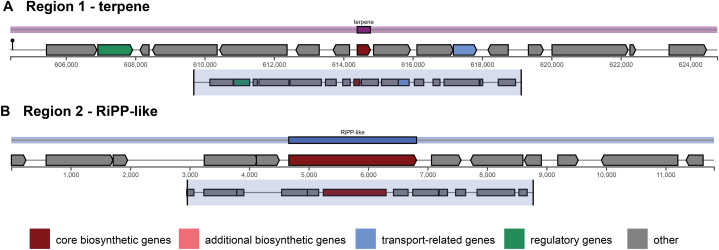
Prediction of genes associated with secondary metabolite biosynthesis in *L. fermentum* SD7 using antiSMASH.

Two potential gene clusters involved in secondary metabolite biosynthesis were identified in strain SD7 using the bioinformatics tool antiSMASH (Fig. 7). The first region spans from 604,397 to 624,777 bp in the chromosome and was identified as a terpene cluster. Within this cluster, three functions were reported in each locus: ctg1_581, ctg1_587, and ctg1_590, which were predicted to be associated with regulatory, biosynthetic, and transport functions, respectively. The second region, spanning from 1 to 11,813 bp in the plasmid, was identified as a RiPP-like region, containing only a single locus, ctg5_6, encoding a biosynthetic function. An overview of the secondary metabolite biosynthetic gene clusters is presented in Table 2.

**Table 2:** Prediction of secondary metabolite biosynthetic gene clusters in *L. fermentum* SD7.

Contig	Region		Type	Locus	Length		Description
	Start bp	End bp			NT	AA	
Contig1	604,397	624,777	terpene	ctg1_581	1002	333	regulatory
				ctg1_587	381	126	biosynthetic
				ctg1_590	672	223	transport
Contig P5	1	11,813	RiPP-like	ctg5_6	2148	715	biosynthetic

## 4 Discussion

Genotypic differences confer specific beneficial properties to individual probiotic strains, which exhibit different potential uses in various fields, such as food manufacturing, human health, and the biotechnology industry. Investigating the genomes of probiotic strains may help predict their properties and enable selection of appropriate potential strains. In this study, we investigated the genome of *L. fermentum* SD7 using next-generation sequencing and several bioinformatic tools. Integrating these techniques allows for a comprehensive evaluation of bacterial strains' safety prior to their use in food production or probiotic applications. Results from *in silico* safety assessments offer crucial insights, guiding further experimental research to confirm the strains' safety and uncover any potential risks. The strains exhibited probiotic properties by demonstrating acid tolerance, bile salt tolerance, and cold shock responses. These findings suggest that the strains could be beneficial for gastrointestinal health and may enhance survival in challenging environments [57]. Further experimental validation will be essential to establish their efficacy and safety for use in probiotic applications.

We identified the core genome comprised of 910 genes, and comparison with related strains FS-10 and L13 revealed 91 strain-specific genes. We identified four putative CRISPR repeats in strain SD7, whereas strains FS-10 and L13 lacked a detectable CRISPR-Cas system. A previous study investigating CRISPRs and Cas genes in 224 *L. fermentum* strains identified at least one CRISPR gene in the genome of 210 *L. fermentum* strains, and Cas genes in the genome of 159 *L. fermentum* strains, which demonstrates the diversity of *L. fermentum* species in terms of both CRISPR systems and genomic rearrangements [58]. In probiotics, CRISPR-Cas systems can play a protective role by preventing horizontal gene transfer of undesirable genes, such as those associated with virulence or antimicrobial resistance, thus enhancing strain safety for therapeutic and food applications. The genomic stability conferred by CRISPR in SD7 potentially reduces the risk of acquiring and propagating genes that could compromise probiotic safety, further supporting its suitability for human use [59]. We therefore hypothesized that *L. fermentum* SD7 has been exposed to a wider range of habitats than originally thought and has undergone several evolutionary processes as each strain optimized for its niche, thereby leading to the high level of diversity.

*L. fermentum* SD7 exhibited a 51.49 % GC content, comparable to the 49–56 % GC content of typical *L. fermentum* strains, including strains 3872, CECT5716, F-6, IFO3956, JDFM216, NCC2970, SNUV175, YLF016, AGR1485, HFD1, and SK152 [[60], [61], [62], [63], [64], [65], [66]]. More than 40 % of the genes code for unknown or hypothetical proteins, indicating that additional examination of the *L. fermentum* SD7 genome is necessary. We identified three prophage genes in the genome, which may enable *Limosilactobacillus* species to adapt to diverse habitats, including those with varying nutritional requirements or stress conditions such as sugar fermentation, limited nutrient availability, and bile salt tolerance [67,68]. Given the significance of ARGs and virulence genes in health and biotechnological applications, we investigated whether *L. fermentum* SD7 contains any predicted ARGs or virulence genes. None were detected, supporting the safe designation of strain SD7 under GRAS or QPS (Qualified Presumption of Safety) status, making it suitable for use as a food additive or in other healthcare applications.

After examining the genome of *L. fermentum* SD7, we conducted a comparative genomic analysis with 153 other strains sourced from the NCBI database. Six clades were delineated for *L. fermentum*, with strain SD7 falling within clade 4, which encompassed 58 *L. fermentum* strains. Core genome SNP analysis revealed that strains FS-10 and L13 share a close relationship with strain SD7, sharing the majority of core genes. Both FS-10 and L13 were isolated from human feces, while SD7 originated from the human oral cavity. However, the SD7 genome was larger than that of the other strains, possibly due to the higher number of identified plasmid contigs in the SD7 genome compared to FS-10 and L13. The plasmids in *L. fermentum* SD7 harbor a diverse array of genes involved in crucial cellular functions such as DNA maintenance, gene regulation, and antimicrobial resistance, indicating strain's adaptability and potential for survival in various environments.

Pan-genome analysis based on core gene SNPs of 154 *L. fermentum* genomes, including strain SD7, revealed significant differences in the proportion of core and accessory genes among the collected strains (Fig. 5), indicating a limited number of conserved genes in *L. fermentum* spp. Of the 12,598 genes from the 154 genomes, 10,169 (81 %) were identified as accessory genes, indicating significant variation and diversity, which may be attributed to the ecological versatility of *L. fermentum*. Several genes associated with sugar fermentation are responsible for the adaptation of *L. fermentum* to specific habitats [58]. Moreover, the number of pan-genes in *L. fermentum* continued to increase with an increasing number of analyzed genomes, implying open-genome characteristics for this species. There is therefore a high probability of identifying new strains of *L. fermentum* in various environments. The functional differences of strain-specific genes among different strains of *L. fermentum* were investigated based on COG assignment. As a result, we found that the high abundance of strain-specific genes in all strains was not categorized into any COGs, reflecting the insufficient COG classification to cover these genes with novel functions. In the COG category of nucleotide transport and metabolism, the proportion of strain-specific genes in strain FS-10 was overrepresented compared to L13 and SD7. This indicates that FS-10 may have a high level of nucleotide synthesis promoting its growth and development in the human environment, as this strain was also isolated from human-related samples [69]. It is also worth noting that the COG functional group T (signal transduction) of FS-10 contained a significantly higher number of strain-specific genes than L13 (p = 0.025), facilitating its response and adaptation to human host environments [70].

*Lactobacillus* are potential probiotics readily internalized by host or pathogen cells [29,71], which promotes host defense mechanisms by eliminating pathogenic adherence or facilitating auto-aggregation of related species [72,73]. After examining the genome of *L. fermentum* SD7 and compared its genomic and functional features with those of widely used probiotic strains, such as *L. rhamnosus* GG, *L. plantarum* WCFS1, and *L. acidophilus* NCFM, to highlight its unique advantages. Unlike those species, which possess specific genes for immune modulation and biofilm formation, *L. fermentum* SD7 demonstrated distinctive genomic attributes related to adhesion and aggregation, with 25 putative genes associated with these functions. These include the inner membrane protein YagU (*yugU*). This gene is part of the pathogenicity island of uropathogenic *Escherichia coli* and encodes fimbrial adhesin [74]. Additionally, the membrane protein YdfK (*ydfK*) and inner membrane transporter YicL (*yicL*) found in SD7, also have immunomodulatory capacity through adhesion to mucins and epithelial cells [75].

Lipoproteins are important components that support inner membrane integrity and promote cell adhesion [76,77]. In this study, we identified two lipoprotein-associated genes, *tpn32* (membrane lipoprotein TpN32) and *lspA* (lipoprotein signal peptidase). In Gram-positive bacteria, lipoproteins support bacterial survival under diverse conditions by modulating nutrient uptake, cell wall metabolism, cell division, transmembrane signal, and adhesion to host tissues [78]. Another important property of LAB is glutaglucan production [79]. In SD7, we identified two UDP-N-acetylglucosamine-peptide N-acetylglucosaminyltransferase genes (*gtfA_1* and *gtfA_2*) and a glucosyltransferase-SI gene (*gtfC*) likely involved in glucan synthesis [80]. Several adhesion and aggregation-associated genes were also identified in SD7. *eno2*, an enolase gene, regulates metabolic enzymes that serve as virulence factors in pathogens but also mediates adhesion in probiotics [81]. *tpiA*, linked to adhesion and pathogen exclusion, is commonly found in probiotic species [82]. Biofilm formation, an important probiotic trait in species such as *L. rhamnosus* and *L. fermentum*, relies on fimbriae [83,84]. In this study, we identified *fimA* and *mltF* genes associated with pili and fimbriae production on the bacterial surface and host epithelial adhesion, which are critical for establishing biofilms. By forming biofilms, probiotics can inhibit pathogen colonization through competitive exclusion, thereby enhancing their survival and efficacy in the gut [85,86]. Additionally, SD7 contains an EPS gene cluster on its chromosome, similar to clusters in related strains, such as *L. fermentum* YL-11, *L. bulgaricus* Lfi5, *L. fermentum* TDS030603, *L. paraplantarum* BGCG11, *L. rhamnosus* GG, and *L. paracasei* BGSJ2-8 [56,[87], [88], [89], [90], [91]]. The cluster may enhance adhesion and aggregation, facilitating host colonization and pathogen exclusion [22]. Internalization, adhesion, and aggregation enhance the effectiveness of probiotic strains by inhibiting pathogen invasion of intestinal epithelial cells, either through auto-aggregation to form a barrier or co-aggregation with pathogens [92,93]. These traits suggest that SD7 may have superior potential for forming protective barriers on mucosal surfaces, a crucial attribute in both food and therapeutic probiotics.

The identification of two gene clusters involved in secondary metabolite biosynthesis in strain SD7, as detected by antiSMASH, suggests a potential for health-beneficial compounds. The terpene biosynthetic cluster, with its regulatory (ctg1_581), biosynthetic (ctg1_587), and transport (ctg1_590) functions, indicates a coordinated mechanism for terpene production, which may contribute anti-inflammatory, antioxidant, and antimicrobial benefits, supporting gut health and pathogen resistance [94]. Additionally, the RiPP-like gene cluster (ctg5_6) may produce ribosomally synthesized peptides with antimicrobial activity, aiding pathogen inhibition and promoting microbiome balance [95].

The findings of this study offer a comprehensive genomic and functional profile of *L. fermentum* SD7, revealing distinct attributes that underscore its probiotic potential. By identifying unique genes associated with adhesion, aggregation, and immune modulation, this research enhances our understanding of SD7's functionality. The identification of a complex EPS gene cluster, CRISPR-Cas system, and two potential secondary metabolite gene clusters underlines SD7's adaptability and versatility, making it a robust candidate for therapeutic probiotic applications. This innovative approach to characterizing SD7's genome is significant, as it not only establishes a genomic basis for its probiotic functions but also provides insights into its use in human health applications where biofilm formation, pathogen exclusion, and immune modulation are desired properties.

## 5 Conclusion

The genome analysis of *L. fermentum* SD7 highlights its potential as a safe and effective probiotic strain. Safety attributes include genome stability, supported by the CRISPR-Cas system, which could prevent horizontal transfer of undesirable genes. For survival ability, stress-response genes likely contribute to SD7's resilience in various environments, including the human gut. The strain also features secondary metabolite gene clusters—specifically, terpene and RiPP-like clusters—that may provide antimicrobial and anti-inflammatory benefits. Genes related to cell adhesion and aggregation were identified, reinforcing SD7's strong colonization ability, which is critical for pathogen inhibition and interaction with host cells. Collectively, these properties support *L. fermentum* SD7 as a promising candidate for probiotic use in foods or beverages, potentially improving gut health and microbiota balance.

## CRediT authorship contribution statement

**Thunchanok Yaikhan:** Writing – review & editing, Writing – original draft, Software, Methodology, Investigation, Formal analysis, Data curation. **Monwadee Wonglapsuwan:** Writing – original draft, Methodology, Investigation, Funding acquisition, Data curation, Conceptualization. **Nuntiya Pahumunto:** Resources, Methodology, Formal analysis, Data curation. **Natakorn Nokchan:** Methodology, Investigation, Formal analysis. **Rawee Teanpaisan:** Writing – review & editing, Writing – original draft, Supervision, Resources, Methodology, Investigation, Conceptualization. **Komwit Surachat:** Writing – review & editing, Writing – original draft, Validation, Supervision, Software, Project administration, Investigation, Formal analysis, Data curation, Conceptualization.

## Funding

This research was funded by a Postdoctoral Fellowship from 10.13039/501100004508Prince of Songkla University, Thailand, and also received support from the NSRF through the Program Management Unit for Human Resources & Institutional Development, Research and Innovation under grant number B13F660074.

## Declaration of competing interest

The authors declare that they have no known competing financial interests or personal relationships that could have appeared to influence the work reported in this paper.
