# Supplementary material for: Probiogenomic analysis of Limosilactobacillus fermentum SD7, a probiotic candidate with remarkable aggregation abilities
Source: Heliyon. 2025 Feb 3;11(3):e42451. doi: 10.1016/j.heliyon.2025.e42451 (PMC11850171; doi:10.1016/j.heliyon.2025.e42451)
Supplement: Multimedia component 1 [file mmc1.docx]

**Supplementary data**

**Supplementary Figure 1** Percentage of completeness of core genes in the BUSCO analysis for 154 assemblies from the NCBI Reference Sequence Database (RefSeq) and the SD7 assembly obtained in this study.


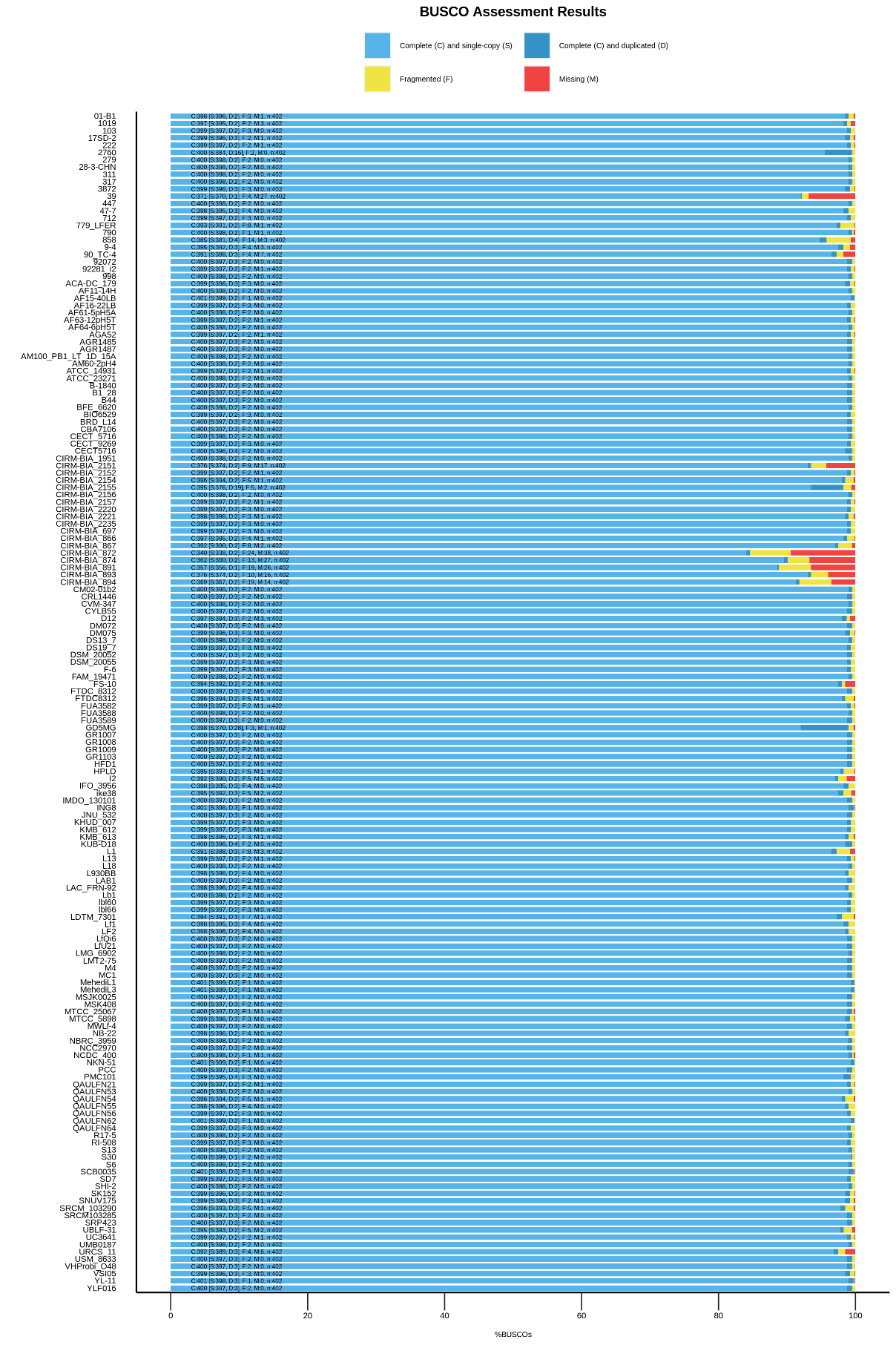


**Supplementary Figure 2** Percentage of completeness of core genes in the BUSCO analysis for the remaining 154 assemblies after filtering out genome sequences with completeness levels lower than 97%.


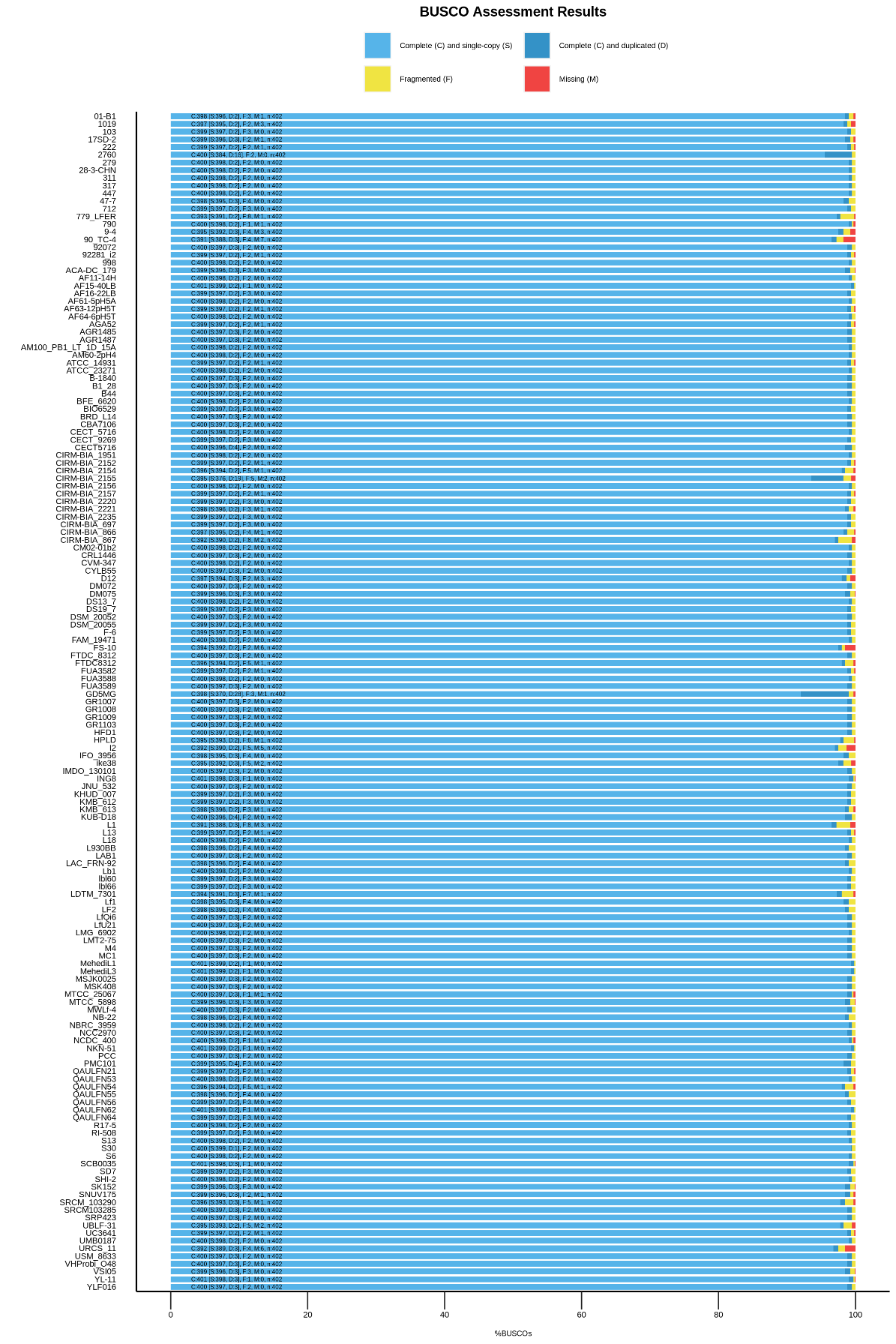


**Supplementary Table 1.** Plasmid contigs identified in the SD7 genome

| Contig | Identification | Query cover | Similarity | Accession |
| --- | --- | --- | --- | --- |
| P1 | *Lactobacillus salivarius* CECT 5713 plasmid pHN1 | 16% | 99% | CP002035.1 |
| P2 | *Limosilactobacillus fermentum* strain SNUV175 plasmid pSNU175-3 | 99% | 99% | CP019032.1 |
| P3 | *Lactiplantibacillus plantarum* strain MWLp-12 plasmid pMWLp-12A | 81% | 97% | CP116750.1 |
| P4 | *Limosilactobacillus fermentum* strain LMT2-75 plasmid p1 | 6% | 98% | CP034100.1 |
| P5 | *Limosilactobacillus fermentum* strain DR9 plasmid unnamed1 | 24% | 98% | CP033372.1 |
| P6 | *Limosilactobacillus reuteri* strain AM_LB1 plasmid unnamed2 | 16% | 99% | CP104441.1 |
| P7 | *Limosilactobacillus fermentum* strain DR9 plasmid unnamed1 | 100% | 98% | CP033372.1 |
| P8 | *Lactiplantibacillus plantarum* strain K25 plasmid unnamed1, complete sequence | 88 | 98% | CP020094.1 |
| P9 | *Limosilactobacillus fermentum* strain SNUV175 plasmid pSNU175-3 | 100 | 99% | CP019032.1 |
| P10 | *Lactiplantibacillus plantarum* strain MWLp-12 plasmid pMWLp-12D | 100 | 98% | CP116753.1 |
| P11 | *Limosilactobacillus fermentum* strain SNUV175 plasmid pSNU175-3 | 100 | 99% | CP019032.1 |
| P12 | *Limosilactobacillus fermentum* strain DR9 plasmid unnamed1 | 100% | 99% | CP033372.1 |
| P13 | *Limosilactobacillus fermentum* strain SNUV175 plasmid pSNU175-3 | 100 | 98% | CP019032.1 |

**Supplementary Table 2.** Genes related to probiotic properties in *L. fermentum* SD7

| Acid tolerance | Function/description |
| --- | --- |
| *arcD*, *arcC1*, *arcA*, *arcD1* | Arginine deiminase pathway components, buffer cells against acidic conditions. |
| *groS, groL* | Chaperonins that assist in protein folding under stress. |
| *clpC_1, clpC_2, clpP_1, clpP_2, clpP_3, clpE, clpX, clpC1* | Protein quality control under stress, which aids acid tolerance. |
| *dnaK, dnaJ* | Chaperone proteins that assist in protein folding under stress conditions. |
| *rutB* | Involved in pyrimidine metabolism, may play a role in managing intracellular pH, aiding acid tolerance. |
| *pucG_1, pucG_2* | Part of purine metabolism, aiding cellular survival under stress, potentially acid tolerance. |
| Bile salt tolerance |  |
| *clcB* | Encodes a chloride channel protein, aiding in ionic homeostasis under bile salt stress. |
| *mprF* | Modifies cell membrane lipids, enhancing resistance to bile salts and antimicrobial peptides. |
| *yhdG_1, yhdG_2* | Involved in lipid metabolism and membrane integrity, supporting survival in bile conditions. |
| *fabZ, fabH, fabD, fabG, fabF, fabI* | Key components in fatty acid biosynthesis, essential for membrane integrity in bile. |
| *ltaS1_1, ltaS1_2* | Lipoteichoic acid synthases, involved in membrane synthesis and stability, aiding bile resistance. |
| *mprA* | Regulatory protein involved in cell wall stress response, maintaining membrane integrity under bile stress. |
| *drrA* | Associated with drug resistance; may play a role in membrane transport, indirectly supporting bile tolerance. |
| Cold shock proteins |  |
| *cspLA* | A cold shock protein that assists in survival during rapid temperature drops. |
| *csp* | Another cold shock protein that helps stabilize RNA and proteins under cold conditions. |
| Stress-Related Genes |  |
| *trxA_1, trxA_2, trxA_3, trxB* | Thioredoxin and thioredoxin reductase variants that protect cells from oxidative damage. |
| *dltA, dltC, dltD* | Involved in the D-alanylation of lipoteichoic acids, strengthening the cell wall under stress. |
| *gltT* | Glutamate transporter, may aid in stress adaptation, especially under nutrient limitation. |
| *ntpJ* | Involved in nucleotide metabolism, potentially contributing to cellular repair under stress. |

**Supplementary Table 3.** Genes carried by plasmid contigs

| Contig | Start | Stop | Strand | Product |
| --- | --- | --- | --- | --- |
| P1 | 3562 | 6930 | + | Chromosome partition protein Smc |
| P1 | 15192 | 16412 | + | IS256 family transposase ISCARN68 |
| P1 | 29311 | 30435 | + | Tyrosine recombinase XerC |
| P1 | 30450 | 31670 | - | IS256 family transposase ISCARN68 |
| P1 | 31886 | 32752 | + | Daunorubicin/doxorubicin resistance ATP-binding protein DrrA |
| P2 | 1085 | 1939 | + | IS3 family transposase ISEnfa5 |
| P2 | 4416 | 4859 | + | putative HTH-type transcriptional regulator |
| P2 | 9323 | 10654 | - | putative pyridine nucleotide-disulfide oxidoreductase RclA |
| P2 | 11145 | 11705 | + | Serine recombinase PinR |
| P2 | 12284 | 13162 | + | IS3 family transposase ISBce13 |
| P2 | 13561 | 13992 | - | Putative universal stress protein |
| P2 | 13992 | 15584 | - | Divalent metal cation transporter MntH |
| P2 | 17451 | 18305 | + | IS3 family transposase ISEnfa5 |
| P2 | 25842 | 26267 | + | DNA topoisomerase 3 |
| P2 | 26307 | 27212 | + | DNA topoisomerase 3 |
| P2 | 28471 | 29658 | + | IS256 family transposase IS1310 |
| P2 | 30836 | 31714 | - | Histidine protein kinase SaeS |
| P2 | 31858 | 32559 | - | Response regulator SaeR |
| P3 | 6527 | 6949 | - | putative protein YjdF |
| P3 | 7927 | 8814 | - | Vitamin B12 import ATP-binding protein BtuD |
| P3 | 10203 | 11552 | + | putative pyridine nucleotide-disulfide oxidoreductase RclA |
| P3 | 16520 | 17104 | + | Putative transposon Tn552 DNA-invertase bin3 |
| P4 | 106 | 1455 | - | Succinyl-diaminopimelate desuccinylase |
| P4 | 1469 | 2674 | - | putative protein |
| P4 | 4601 | 5785 | - | putative protein |
| P4 | 5816 | 6694 | - | Oligopeptide transport ATP-binding protein OppF |
| P4 | 6687 | 7745 | - | Oligopeptide transport ATP-binding protein OppD |
| P4 | 7746 | 8696 | - | Dipeptide transport system permease protein DppC |
| P4 | 8698 | 9630 | - | Oligopeptide transport system permease protein OppB |
| P4 | 9713 | 11341 | - | Dipeptide-binding protein DppE |
| P4 | 12373 | 13221 | + | IS3 family transposase ISSth1b |
| P4 | 13281 | 13652 | + | IS66 family transposase ISCde1 |
| P4 | 13722 | 16448 | - | DNA replication and repair protein RecF |
| P4 | 18135 | 18908 | - | RNA polymerase sigma factor RpoS |

**Supplementary Table 3.** Genes carried by plasmid contigs (cont)

| Contig | Start | Stop | Strand | Product |
| --- | --- | --- | --- | --- |
| P5 | 3244 | 4143 | + | db_xref=COG:COG1192 |
| P5 | 4121 | 4507 | + | Sporulation initiation inhibitor protein Soj |
| P5 | 4666 | 6813 | + | Lactococcin-G-processing and transport ATP-binding protein LagD |
| P5 | 7722 | 8600 | - | IS3 family transposase ISBce13 |
| P5 | 9920 | 11206 | - | ISL3 family transposase ISL3 |
| P5 | 11670 | 11909 | - | Transposon Tn3 resolvase |
| P6 | 379 | 2067 | + | Acetolactate synthase large subunit |
| P6 | 2097 | 3116 | + | Ketol-acid reductoisomerase (NADP(+)) |
| P6 | 3256 | 4503 | + | L-threonine dehydratase biosynthetic IlvA |
| P6 | 4516 | 5307 | + | putative ABC transporter ATP-binding protein YlmA |
| P6 | 5868 | 6338 | + | Transcriptional repressor CopY |
| P6 | 6363 | 7280 | + | Beta-glucoside kinase |
| P6 | 7677 | 8543 | - | Di-/tripeptide transporter |
| P6 | 8630 | 8836 | - | Di-/tripeptide transporter |

**Supplementary Table 4.** Metadata of 154 *L. fermentum* from this study and the RefSeq database

| **Accession** | **Strain** | **Assembly Status** | **Bioproject ID** | **Biosample ID** | **Coverage** | **N50** | **Isolation source** |
| --- | --- | --- | --- | --- | --- | --- | --- |
| GCF_016861465.1 | 01-B1 | Contig | PRJDB11003 | SAMD00270997 | 100 | 102808 | missing |
| GCF_025185835.1 | 1019 | Contig | PRJNA503586 | SAMN10365689 | 195 | 29970 | Food and beverage |
| GCF_002794375.1 | 103 | Contig | PRJNA417365 | SAMN07985466 | 150 | 45848 | Human related specimen |
| GCF_025780195.1 | 17SD-2 | Scaffold | PRJNA888851 | SAMN31222265 | 263 | 45579 | Food and beverage |
| GCF_001368755.1 | 222 | Contig | PRJEB5182 | SAMEA3158475 | 104 | 55702 | missing |
| GCF_008802915.1 | 2760 | Complete | PRJNA566331 | SAMN12786154 | 98.6 | 2267305 | Food and beverage |
| GCF_002794275.1 | 279 | Contig | PRJNA417365 | SAMN08014151 | 150 | 44731 | Human related specimen |
| GCF_002794315.1 | 311 | Contig | PRJNA417365 | SAMN08014154 | 150 | 43265 | Human related specimen |
| GCF_009362835.1 | 317 | Contig | PRJNA578299 | SAMN13056196 | 58.8 | 43204 | Food and beverage |
| GCF_025211175.1 | 447 | Scaffold | PRJNA503586 | SAMN10365681 | 158 | 28605 | Food and beverage |
| GCF_001854105.1 | 47-7 | Complete | PRJNA347617 | SAMN05893390 | 411.1 | 2098685 | missing |
| GCF_025186015.1 | 712 | Contig | PRJNA503586 | SAMN10365685 | 310 | 37680 | Food and beverage |
| GCF_001077025.1 | 779_LFER | Scaffold | PRJNA267549 | SAMN03197989 | 13 | 21226 | missing |
| GCF_025185985.1 | 790 | Contig | PRJNA503586 | SAMN10365687 | 285 | 32087 | Food and beverage |
| GCF_001010245.1 | 90 TC-4 | Contig | PRJNA279953 | SAMN03452288 | 250 | 36919 | missing |
| GCF_028462685.1 | 92072 | Contig | PRJNA926949 | SAMN32886978 | 78 | 24975 | Food and beverage |
| GCF_028462585.1 | 92281_i2 | Contig | PRJNA926949 | SAMN32886984 | 67 | 40285 | Food and beverage |
| GCF_018732245.1 | 9-4 | Complete | PRJNA721242 | SAMN18711544 | 533.86 | 2085632 | Food and beverage |
| GCF_025185825.1 | 998 | Contig | PRJNA503586 | SAMN10365688 | 320 | 34412 | Food and beverage |
| GCF_027686025.1 | AF11-14H | Scaffold | PRJNA903559 | SAMN31807818 | 100 | 61952 | missing |
| GCF_003465085.1 | AF11-4-H | Scaffold | PRJNA482748 | SAMN09734212 | 100 | 45868 | Human related specimen |
| GCF_003464285.1 | AF15-40LB | Scaffold | PRJNA482748 | SAMN09734289 | 100 | 62108 | Human related specimen |
| GCF_003462755.1 | AF16-22LB | Scaffold | PRJNA482748 | SAMN09734308 | 100 | 43257 | Human related specimen |
| GCF_027681265.1 | AF61-5pH5A | Scaffold | PRJNA903559 | SAMN31808042 | 100 | 46474 | missing |
| GCF_027681185.1 | AF63-12pH5T | Scaffold | PRJNA903559 | SAMN31808046 | 100 | 54038 | missing |
| GCF_027681055.1 | AF64-6pH5T | Scaffold | PRJNA903559 | SAMN31808054 | 100 | 38854 | missing |
| GCF_021582915.1 | AGA52 | Complete | PRJNA797952 | SAMN25042641 | 296 | 2001184 | Food and beverage |
| GCF_011032765.1 | AGR1485 | Complete | PRJNA588334 | SAMN13241836 | 989.2 | 2226862 | Human related specimen |
| GCF_011032745.1 | AGR1487 | Complete | PRJNA596816 | SAMN13639333 | 1510.51 | 1939032 | Human related specimen |
| GCF_027661185.1 | AM100_PB1 | Scaffold | PRJNA903559 | SAMN31808592 | 100 | 30116 | missing |
| GCF_027668665.1 | AM60-2pH4 | Scaffold | PRJNA903559 | SAMN31809206 | 100 | 49509 | missing |
| GCF_005341425.1 | B1 28 | Complete | PRJNA477598 | SAMN09475314 | 342 | 1905587 | Food and beverage |
| GCF_030770375.1 | B-1840 | Complete | PRJNA1003957 | SAMN36918642 | 1 | 1907047 | missing |
| GCF_016617695.1 | B44 | Complete | PRJNA689682 | SAMN17214540 | 772 | 2110953 | Food and beverage |
| GCF_002204495.1 | BFE 6620 | Scaffold | PRJNA390617 | SAMN07246874 | 242 | 35982 | Food and beverage |
| GCF_008868475.1 | BIO6529 | Contig | PRJNA574342 | SAMN12856542 | 106.3 | 36893 | Human related specimen |
| GCF_003255875.1 | CBA7106 | Complete | PRJNA390215 | SAMN07224244 | 270 | 2042277 | Human related specimen |
| GCF_000210515.1 | CECT 5716 | Complete | PRJNA43533 | SAMN02604100 | 605 | 2100449 | Human related specimen |
| GCF_900290185.1 | CECT 9269 | Contig | PRJEB24713 | SAMEA104569778 | 377 | 42592 | missing |
| GCF_025191825.1 | CIRM-BIA 1951 | Contig | PRJNA503586 | SAMN10365384 | 101 | 26281 | Food and beverage |
| GCF_025191565.1 | CIRM-BIA 2152 | Contig | PRJNA503586 | SAMN10365397 | 288 | 32843 | Human related specimen |
| GCA_025191655.1 | CIRM-BIA 2153 | Contig | PRJNA503586 | SAMN10365393 | 259 | 3303 | Food and beverage |
| GCF_025191635.1 | CIRM-BIA 2154 | Contig | PRJNA503586 | SAMN10365392 | 138 | 25390 | Food and beverage |
| GCF_025191645.1 | CIRM-BIA 2155 | Scaffold | PRJNA503586 | SAMN10365391 | 105 | 1732905 | Food and beverage |
| GCF_025191725.1 | CIRM-BIA 2156 | Contig | PRJNA503586 | SAMN10365387 | 225 | 22579 | Food and beverage |
| GCF_025191625.1 | CIRM-BIA 2157 | Contig | PRJNA503586 | SAMN10365394 | 360 | 34266 | Food and beverage |
| GCF_025122575.1 | CIRM-BIA 2220 | Contig | PRJNA503586 | SAMN10365214 | 193 | 32658 | Food and beverage |
| GCF_025122475.1 | CIRM-BIA 2221 | Contig | PRJNA503586 | SAMN10365213 | 102 | 30508 | Food and beverage |
| GCF_025191755.1 | CIRM-BIA 2235 | Contig | PRJNA503586 | SAMN10365389 | 284 | 24736 | Food and beverage |
| GCF_025191705.1 | CIRM-BIA 697 | Contig | PRJNA503586 | SAMN10365390 | 417 | 24818 | Food and beverage |
| GCF_025191505.1 | CIRM-BIA 866 | Contig | PRJNA503586 | SAMN10365400 | 215 | 29445 | Human related specimen |
| GCF_025191545.1 | CIRM-BIA 867 | Contig | PRJNA503586 | SAMN10365398 | 295 | 20698 | Human related specimen |
| GCF_027696965.1 | CM02-01b2 | Scaffold | PRJNA903559 | SAMN31809578 | 100 | 73229 | missing |
| GCF_002899655.1 | CRL1446 | Contig | PRJNA429654 | SAMN08355059 | 670 | 75620 | Food and beverage |
| GCF_036281435.1 | CUL67 | Contig | PRJNA482518 | SAMN09708456 | 39.06 | 39274 | Food and beverage |
| GCF_011290755.1 | CVM-347 | Scaffold | PRJNA610980 | SAMN14329897 | 244.7 | 40189 | missing |
| GCF_029537335.1 | CYLB55 | Complete | PRJNA945331 | SAMN33777524 | 74 | 2093653 | Food and beverage |
| GCF_009914845.1 | D12 | Scaffold | PRJNA388578 | SAMN10319712 | 156 | 164687 | Food and beverage |
| GCF_024054255.2 | DM072 | Complete | PRJNA852394 | SAMN29328592 | 518.3 | 2060072 | Human related specimen |
| GCF_024204625.1 | DM075 | Complete | PRJNA853106 | SAMN29360653 | 259.2 | 2204022 | Human related specimen |
| GCF_003710225.1 | DR9 | Complete | PRJNA482260 | SAMN09701875 | 147.94 | 1285454 | Food and beverage |
| GCF_003061865.1 | DS13_7 | Contig | PRJNA336518 | SAMN05942013 | 67.4 | 38640 | Supplement |
| GCF_003053105.1 | DS19_7 | Contig | PRJNA336518 | SAMN05942014 | 49.4 | 43520 | Supplement |
| GCF_013394085.1 | DSM 20052 | Complete | PRJNA545488 | SAMN11893902 | 125 | 1887974 | Food and beverage |
| GCF_001436835.1 | DSM 20055 | Scaffold | PRJNA222257 | SAMN02797777 | 100 | 43184 | missing |
| GCF_000397165.1 | F-6 | Complete | PRJNA49143 | SAMN02603935 | 100 | 2064620 | missing |
| GCF_005864145.1 | FAM 19471 | Contig | PRJNA543085 | SAMN11653939 | 62 | 34842 | Food and beverage |
| GCF_029606345.1 | FS-10 | Scaffold | PRJNA924529 | SAMN32759542 | 100 | 54013 | Human related specimen |
| GCF_002119645.1 | FTDC 8312 | Complete | PRJNA382300 | SAMN06703219 | 146 | 2239921 | Human related specimen |
| GCF_000417005.1 | FTDC8312 | Scaffold | PRJNA205894 | SAMN02469912 | 90 | 37977 | Human related specimen |
| GCF_020844625.1 | FUA3582 | Scaffold | PRJNA526935 | SAMN21223652 | 400 | 45570 | Food and beverage |
| GCF_004683835.1 | FUA3588 | Scaffold | PRJNA526935 | SAMN11109659 | 2000 | 57818 | Food and beverage |
| GCF_004683795.1 | FUA3589 | Scaffold | PRJNA526935 | SAMN11119341 | 2000 | 37233 | Food and beverage |
| GCF_028216035.1 | GD5MG | Complete | PRJNA925011 | SAMN32778798 | 2912.48 | 1834134 | missing |
| GCF_017134375.1 | GR1007 | Complete | PRJNA702613 | SAMN17976236 | 105.6 | 2238385 | Food and beverage |
| GCF_017134355.1 | GR1008 | Complete | PRJNA702613 | SAMN17976237 | 115.3 | 2237764 | Food and beverage |
| GCF_017134335.1 | GR1009 | Complete | PRJNA702613 | SAMN17976238 | 101.5 | 2227144 | Food and beverage |
| GCF_017068315.1 | GR1103 | Complete | PRJNA702615 | SAMN17976243 | 105 | 2234899 | Supplement |
| GCF_001297025.1 | HFB3 | Contig | PRJNA294944 | SAMN04038470 | 75 | 735712 | Human related specimen |
| GCF_012273035.1 | HFD1 | Complete | PRJNA615901 | SAMN14470504 | 1100 | 2101878 | missing |
| GCF_014830195.1 | HPLD | Contig | PRJNA479556 | SAMN09580242 | 56 | 25241 | Human related specimen |
| GCF_009295755.1 | I2 | Scaffold | PRJNA577465 | SAMN13031766 | 575 | 39037 | Food and beverage |
| GCF_016861955.1 | ike38 | Complete | PRJDB10981 | SAMD00268105 | 550 | 1973978 | missing |
| GCF_900205745.1 | IMDO 130101 | Complete | PRJEB24519 | SAMEA104224050 | 37 | 2089202 | Food and beverage |
| GCF_021128975.1 | ING8 | Contig | PRJNA785757 | SAMN23575918 | 51 | 41442 | Food and beverage |
| GCF_024800585.1 | JNU 532 | Complete | PRJNA872884 | SAMN30472492 | 96 | 2077416 | Food and beverage |
| GCF_024385625.1 | KHUD_007 | Scaffold | PRJNA860016 | SAMN29815206 | 1411 | 44326 | missing |
| GCF_003346325.1 | KMB_612 | Contig | PRJNA474823 | SAMN09398944 | 261 | 34941 | Food and beverage |
| GCF_003346315.1 | KMB_613 | Contig | PRJNA474823 | SAMN09398945 | 212 | 34618 | Food and beverage |
| GCF_023370755.1 | KUB-D18 | Scaffold | PRJNA836376 | SAMN28158841 | 658 | 36068 | Human related specimen |
| GCF_018866265.1 | L1 | Complete | PRJNA735609 | SAMN19589998 | 150 | 2000827 | Food and beverage |
| GCF_012070605.1 | L13 | Scaffold | PRJNA521581 | SAMN10906232 | 100 | 45915 | missing |
| GCF_012070625.1 | L18 | Scaffold | PRJNA521583 | SAMN10906314 | 100 | 40503 | missing |
| GCF_001039735.1 | L930BB | Contig | PRJEB4718 | SAMEA3158477 | 283 | 49484 | missing |
| GCF_021278125.1 | LAB1 | Contig | PRJNA786104 | SAMN23636590 | 15 | 34128 | Food and beverage |
| GCF_002192435.1 | LAC FRN-92 | Complete | PRJNA389240 | SAMN07192734 | 71.2 | 2063606 | Human related specimen |
| GCF_022844685.1 | Lb1 | Scaffold | PRJNA644208 | SAMN15454221 | 110.5 | 42010 | Food and beverage |
| GCF_027857625.1 | lbl60 | Contig | PRJNA918972 | SAMN32612942 | 1310 | 43756 | Food and beverage |
| GCF_027857595.1 | lbl66 | Contig | PRJNA918972 | SAMN32612943 | 870 | 44030 | Food and beverage |
| GCF_003346795.1 | LDTM 7301 | Complete | PRJNA340350 | SAMN05712721 | 409 | 2046196 | Food and beverage |
| GCF_000472265.1 | Lf1 | Contig | PRJNA198062 | SAMN02053534 | 86.9 | 28894 | Human related specimen |
| GCF_003261135.1 | LF2 | Scaffold | PRJNA476494 | SAMN09435684 | 91 | 32695 | Food and beverage |
| GCF_000966835.2 | LfQi6 | Complete | PRJNA276297 | SAMN03372370 | 99 | 2098510 | missing |
| GCF_002869825.2 | LfU21 | Complete | PRJNA417365 | SAMN08290293 | 100 | 2099581 | Human related specimen |
| GCF_029823225.1 | LMG 6902 | Contig | PRJNA933129 | SAMN33225764 | 71 | 40106 | missing |
| GCF_003855655.1 | LMT2-75 | Complete | PRJNA505388 | SAMN10417155 | 270 | 2298221 | Food and beverage |
| GCA_016861505.1 | LT20 | Contig | PRJDB11003 | SAMD00270998 | 639 | 2105249 | missing |
| GCF_023612195.1 | M4 | Complete | PRJNA787400 | SAMN23798833 | 800 | 2032186 | Food and beverage |
| GCF_026930525.1 | MC1 | Scaffold | PRJNA769625 | SAMN22155537 | 400 | 42316 | Human related specimen |
| GCF_002942005.1 | MD IIE-4657 | Contig | PRJNA434637 | SAMN08565625 | 107.8 | 43935 | Food and beverage |
| GCF_029334835.1 | MehediL1 | Contig | PRJNA944999 | SAMN33770425 | 225 | 29159 | Food and beverage |
| GCF_029334875.1 | MehediL3 | Contig | PRJNA945008 | SAMN33770590 | 178 | 23064 | Food and beverage |
| GCF_029691425.1 | MSJK0025 | Complete | PRJNA951508 | SAMN34044604 | 432 | 2114971 | Food and beverage |
| GCF_029223925.1 | MSK408 | Complete | PRJNA941153 | SAMN33591059 | 65 | 2077616 | missing |
| GCF_002356135.1 | MTCC 25067 | Complete | PRJDB5494 | SAMD00073748 | 560 | 1954694 | missing |
| GCF_004208815.1 | MTCC 5898 | Complete | PRJNA520814 | SAMN10868412 | 85 | 2098685 | Human related specimen |
| GCF_028463945.1 | MWLf-4 | Complete | PRJNA914068 | SAMN32317227 | 225 | 2221475 | missing |
| GCF_000496435.1 | NB-22 | Scaffold | PRJNA223167 | SAMN02470787 | 114 | 38875 | missing |
| GCF_006538825.1 | NBRC 3959 | Contig | PRJDB6022 | SAMD00093696 | 96 | 36113 | missing |
| GCF_001742205.1 | NCC2970 | Complete | PRJNA336464 | SAMN05510874 | 250 | 1949874 | missing |
| GCF_002798075.1 | NCDC 400 | Scaffold | PRJNA398455 | SAMN07510693 | 1254 | 27950 | Food and beverage |
| GCF_016918895.1 | NKN-51 | Scaffold | PRJNA701539 | SAMN17885229 | 6 | 1654674 | Food and beverage |
| GCF_024494505.1 | PMC101 | Complete | PRJNA860760 | SAMN29861940 | 277 | 2086671 | Human related specimen |
| GCF_022509425.1 | QAULFN21 | Scaffold | PRJNA744373 | SAMN20114169 | 300 | 219560 | Food and beverage |
| GCF_022509445.1 | QAULFN53 | Scaffold | PRJNA744373 | SAMN20114170 | 300 | 37704 | Food and beverage |
| GCF_022509455.1 | QAULFN54 | Scaffold | PRJNA744373 | SAMN20114171 | 300 | 37977 | Food and beverage |
| GCF_022509385.1 | QAULFN55 | Scaffold | PRJNA744373 | SAMN20114172 | 300 | 38875 | Food and beverage |
| GCF_022509365.1 | QAULFN56 | Scaffold | PRJNA744373 | SAMN20114173 | 300 | 43184 | Food and beverage |
| GCF_022509335.1 | QAULFN62 | Scaffold | PRJNA744373 | SAMN20114174 | 300 | 62108 | Food and beverage |
| GCF_022509325.1 | QAULFN64 | Scaffold | PRJNA744373 | SAMN20114175 | 300 | 43184 | Food and beverage |
| GCF_029872335.1 | R17-5 | Scaffold | PRJNA957267 | SAMN34247966 | 200 | 38634 | Food and beverage |
| GCF_001982185.1 | RI-508 | Scaffold | PRJNA343164 | SAMN05717758 | 30 | 34769 | Food and beverage |
| GCF_900163595.1 | S13 | Contig | PRJEB19182 | SAMEA80310418 | 548 | 50830 | Food and beverage |
| GCF_011420355.1 | S30 | Contig | PRJNA520885 | SAMN10869160 | 100 | 40939 | missing |
| GCF_900163585.1 | S6 | Contig | PRJEB19181 | SAMEA80264668 | 632 | 50830 | Food and beverage |
| GCF_022819245.1 | SCB0035 | Complete | PRJNA818844 | SAMN26885050 | 641.66 | 2016236 | Food and beverage |
| GCF_002591935.1 | SHI-2 | Contig | PRJNA391358 | SAMN07267218 | 10 | 51268 | Human related specimen |
| GCF_002242615.1 | SK152 | Complete | PRJNA318540 | SAMN04858206 | 500 | 2092273 | Food and beverage |
| GCF_001941785.1 | SNUV175 | Complete | PRJNA358449 | SAMN06174220 | 276 | 2176678 | Human related specimen |
| GCF_004063635.1 | SRCM 103290 | Complete | PRJNA514937 | SAMN10738354 | 335.48 | 2120877 | Food and beverage |
| GCF_004063515.1 | SRCM103285 | Complete | PRJNA514921 | SAMN10737529 | 218 | 2146888 | Food and beverage |
| GCF_029223965.1 | SRP423 | Complete | PRJNA941172 | SAMN33591107 | 64.9 | 2077616 | missing |
| GCF_015160755.1 | UBLF-31 | Contig | PRJNA493554 | SAMN15930389 | 824 | 19303 | Human related specimen |
| GCF_014926345.1 | UC3641 | Contig | PRJNA524466 | SAMN11026760 | 1213 | 40290 | missing |
| GCF_001297905.1 | UCO-979C | Contig | PRJNA296497 | SAMN04100088 | 19.46 | 39558 | missing |
| GCF_002863265.1 | UMB0187 | Scaffold | PRJNA316969 | SAMN08193695 | 167.34 | 40979 | missing |
| GCF_024137685.1 | URCS 11 | Contig | PRJNA834610 | SAMN28052912 | 1359.4 | 496276 | Human related specimen |
| GCF_009676625.1 | USM 8633 | Complete | PRJNA312743 | SAMN04531192 | 200 | 2238401 | Food and beverage |
| GCF_024637935.1 | VHProbi O48 | Complete | PRJNA824242 | SAMN27397806 | 804.06 | 2034355 | missing |
| GCA_002072085.1 | VRI-003 | Complete | PRJNA378457 | SAMN06546707 | 600 | 1949297 | Supplement |
| GCF_029011825.1 | VSI05 | Complete | PRJNA934404 | SAMN33275432 | 504.253 | 2215744 | missing |
| GCF_003860425.1 | YL-11 | Complete | PRJNA507070 | SAMN10484493 | 200 | 1908534 | Food and beverage |
| GCF_018884205.1 | YLF016 | Complete | PRJNA682291 | SAMN16987128 | 200 | 2094354 | missing |
| JASNVX000000000 | SD7 | Scaffold | PRJNA977818 | SAMN35534915 | 300 | 2034608 | Human related specimen |

**Supplementary Figure 3.** Heatmap illustrating the average nucleotide identity (ANI) between strain SD7 and typical strains of the genus *Limosilactobacillus*, calculated using OAT software. *Pediococcus pentosaceus* represents the outgroup for comparison.


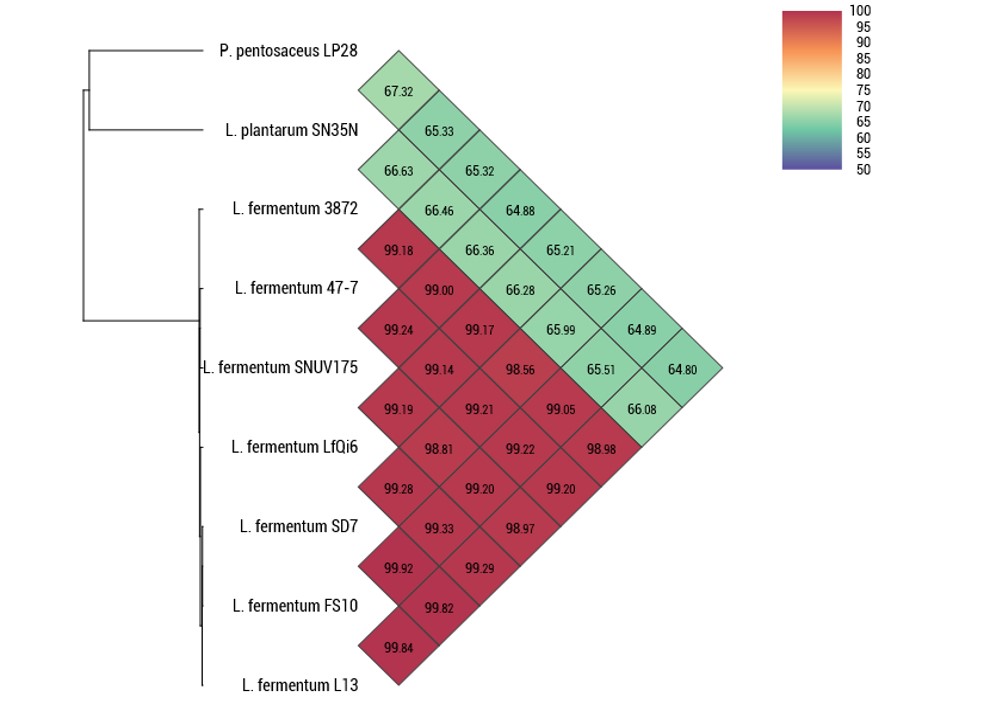


**Supplementary Table 5.** Gene cluster associated with exopolysaccharide in *L. fermentum* SD7

| Interval | Protein Names | Categories | Predicted functions | Gene Names | Organism | Length |
| --- | --- | --- | --- | --- | --- | --- |
| 1,068,154 -> 1,067,585 | Cytokinin riboside 5'-monophosphate phosphoribohydrolase | biosynthesis process | Cytokinin biosynthesis | LACFE_CDS0451 | *L. fermentum* | 170 AA |
| 1,068,661 -> 1,068,296 | DUF4828 domain-containing protein | unknown | Unknown | IV46_GL000139, LACFE_CDS0450 | *L. fermentum* | 108 AA |
| 1,068,826 -> 1,069,836 | Oxidoreductase | nucleotide binding | Nucleotide binding | C0965_008120, C0965_03745, IV46_GL000140, JIO03_07950, LACFE_CDS0449 | *L. fermentum* | 336 AA |
| 1,069,849 -> 1,071,168 | ATP-dependent RNA helicase | nucleotide binding | ATP binding, helicase activity, nucleic acid binding | C1Y38_03120, LACFE_CDS0448 | *L. fermentum* | 442 AA |
| 1,072,257 -> 1,071,823 | Teichoic acid glycosylation protein | biosynthesis process | Polysaccharide biosynthesis | LACFE_CDS0447 | *L. fermentum* | 144AA |
| 1,072,713 -> 1,072,267 | Probabale flavodoxin | transferase | Transferase activity | LACFE_CDS0446 | *L. fermentum* | 150 AA |
| 1,072,826 -> 1,073,683 | Methionine aminopeptidase | metal ion binding | transition metal ion binding, | map, BUW47_07845, C0965_008095, C0965_03720, C1Y38_03140, GC247_03455, IV46_GL000145, JIO03_07920, LACFE_CDS0445, LF130101_1639, LFER_1040 | *L. fermentum* | 285 AA |
| 1,073,840 -> 1,075,537 | Sulfatase N-terminal domain-containing protein | hydrolase activity | Membrane, sulfuric ester hydrolase activity | LACFE_CDS0444 | *L. fermentum* | 979 AA |
| 1,075,525 -> 1,076,250 | Sulfatase N-terminal domain-containing protein | hydrolase activity | Membrane, sulfuric ester hydrolase activity | LACFE_CDS0444 | *L. fermentum* | 979 AA |
| 1,076,272 -> 1,076,487 | Sulfatase N-terminal domain-containing protein | hydrolase activity | Membrane, sulfuric ester hydrolase activity | LACFE_CDS0444 | *L. fermentum* | 979 AA |
| 1,076,616 -> 1,077,512 | YihY/virulence factor BrkB family protein | cellular component | Membrane | LACFE_CDS0443 | *L. fermentum* | 299 AA |

**Supplementary Table 5.** Gene cluster associated with exopolysaccharide *in L. fermentum* SD7

| Interval | Protein Names | Categories | Predicted functions | Gene Names | Organism | Length |
| --- | --- | --- | --- | --- | --- | --- |
| 1,077,908 -> 1,077,693 | DUF2922 domain-containing protein | unknown | Unknown | BUW47_07860, C0965_008080, C0965_03705, C1Y38_03155, DVR01_08040, GC247_03470, GJA14_08725, IV46_GL000148, JIO03_07895, LACFE_CDS0442, LF25067_01560, LFER_1035 | *L. fermentum* | 71 AA |
| 1,078,150 -> 1,077,944 | DUF1659 domain-containing protein | unknown | Unknown | LACFE_CDS0441 | *L. fermentum* | 76 AA |
| 1,078,244 -> 1,079,062 | Regulatory protein RecX | regulation | Regulation of DNA repair | recX, LACFE_CDS0440 | *L. fermentum* | 273 AA |
| 1,079,232 -> 1,079,885 | Exopolysaccharide biosynthesis polyprenyl glycosylphosphotransferase | transferase | Riboflavin biosynthetic process | BUW47_07875, C0965_008065, C0965_03690, C1Y38_03170, GC247_03485, GJA14_08710, IV46_GL000151, JIO03_07880, LACFE_CDS0439 | *L. fermentum* | 217 AA |
| 1,079,897 -> 1,080,658 | Exopolysaccharide biosynthesis protein | biosynthesis process | Exopolysaccharide biosynthesis | LBLM1_02075 | *L. mucosae* LM1 | 256 AA |
| 1,080,669 -> 1,081,892 | Polysaccharide polymerase | cellular component | membrane | PS3_16412 | *L. gastricus* PS3 | 410 AA |
| 1,081,867 -> 1,082,484 | Polysaccharide chain length determinant N-terminal domain-containing protein | biosynthesis process | lipopolysaccharide biosynthetic process | LACFE_CDS0436 | *L. fermentum* | 188 AA |
| 1,082,599 -> 1,083,720 | UDP-galactopyranose mutase | transferase | UDPgalactopyranose mutase activity, trans-hexaprenyltranstransferase activity | glf, C1Y38_03190, DVR01_08075, JIO03_07860, LACFE_CDS0435, LF25067_01549 | *L. fermentum* | 373 AA |
| 1,083,723 -> 1,085,141 | Flippase | cellular component | Plasma membrane | LBLM1_02140 | *L. mucosae* LM1 | 474 AA |

**Supplementary Table 5.** Gene cluster associated with exopolysaccharide in *L. fermentum* SD7

| Interval | Protein Names | Categories | Predicted functions | Gene Names | Organism | Length |
| --- | --- | --- | --- | --- | --- | --- |
| 1,085,151 -> 1,086,209 | Acyltransferase 3 domain-containing protein | transferase | Membrane, acyltransferase activity, transferring groups other than amino-acyl groups | LACFE_CDS0433 | *L. fermentum* | 352 AA |
| 1,086,355 -> 1,088,073 | Mannosyl-glycoprotein endo-beta-N-acetylglucosamidase-like domain-containing protein | hydrolase activity | Amidase activity | LACFE_CDS0432 | *L. fermentum* | 385 AA |
| 1,088,210 -> 1,088,482 | Muramoyltetrapeptide carboxypeptidase | hydrolase activity | muramoyltetrapeptide carboxypeptidase activity | LACFE_CDS0431 | *L. fermentum* | 532 AA |
| 1,088,677 -> 1,089,147 | N-acetylmuramoyl-L-alanine amidase, family 2 | hydrolase activity | N-acetylmuramoyl-L-alanine amidase activity | Lreu_1365 | *L. reuteri* DSM 20016 | 514 AA |
| 1,089,396 -> 1,090,991 | Muramoyltetrapeptide carboxypeptidase | hydrolase activity | muramoyltetrapeptide carboxypeptidase activity | LACFE_CDS0431 | *L. fermentum* | 532 AA |
| 1,091,134 -> 1,091,697 | Dentin sialophosphoprotein | signal peptide | Signal peptide | Lreu_1363 | *L. reuteri* DSM 20016 | 189 AA |
| 1,091,787 -> 1,092,719 | Glycosyl transferase family 2 | transferase | Glycosyl transferase activity | LACFE_CDS0429 | *L. fermentum* | 323 AA |
| 1,092,719 -> 1,093,828 | Permease | transport | Protein transporter | LACFE_CDS0428 | *L. fermentum* | 370 AA |
| 1,093,916 -> 1,094,893 | Glycosyltransferase, group 2 family protein | transferase | Glycosyl transferase activity | LACFE_CDS0427 | *L. fermentum* | 325 AA |
